# Molecular classification and prediction in gastric cancer

**DOI:** 10.1016/j.csbj.2015.08.001

**Published:** 2015-08-13

**Authors:** Xiandong Lin, Yongzhong Zhao, Won-min Song, Bin Zhang

**Affiliations:** a Department of Genetics and Genomic Sciences, Icahn Institute of Genomics and Multiscale Biology, Icahn School of Medicine at Mount Sinai, 1470 Madison Avenue, NY 10029, USA; b Fujian Provincial Key Laboratory of Translational Cancer Medicine, Fujian Provincial Cancer Hospital, No. 420 Fuma Road, Jinan District, Fuzhou, Fujian 350014, PR China

**Keywords:** Gastric cancer, Gene expression profiling, Molecular subtyping, Molecular classification

## Abstract

Gastric cancer, a highly heterogeneous disease, is the second leading cause of cancer death and the fourth most common cancer globally, with East Asia accounting for more than half of cases annually. Alongside TNM staging, gastric cancer clinic has two well-recognized classification systems, the Lauren classification that subdivides gastric adenocarcinoma into intestinal and diffuse types and the alternative World Health Organization system that divides gastric cancer into papillary, tubular, mucinous (colloid), and poorly cohesive carcinomas. Both classification systems enable a better understanding of the histogenesis and the biology of gastric cancer yet have a limited clinical utility in guiding patient therapy due to the molecular heterogeneity of gastric cancer. Unprecedented whole-genome-scale data have been catalyzing and advancing the molecular subtyping approach. Here we cataloged and compared those published gene expression profiling signatures in gastric cancer. We summarized recent integrated genomic characterization of gastric cancer based on additional data of somatic mutation, chromosomal instability, EBV virus infection, and DNA methylation. We identified the consensus patterns across these signatures and identified the underlying molecular pathways and biological functions. The identification of molecular subtyping of gastric adenocarcinoma and the development of integrated genomics approaches for clinical applications such as prediction of clinical intervening emerge as an essential phase toward personalized medicine in treating gastric cancer.

## 1 Introduction

Gastric cancer (GC) is the second leading cause of cancer death and the fourth most prevalent malignancy worldwide, accounting for 8% of cancer incidence and 10% of cancer deaths [1]. In the United States, about 21,000 cases of gastric cancer (61% are men and 39% are women) were diagnosed and about 10,000 patients died from this disease in 2012 [2]. Many factors such as ineffective screening, diagnosis, and treatment approaches contribute to the high incidence and mortality rates of GC [3,4].

Tumor staging has been established and validated as the best predictor of patient survival. Besides tumor node metastasis (TNM) staging, gastric cancer clinic has two well-recognized classification systems, the Lauren classification that subdivides gastric adenocarcinoma into intestine and diffuse types and the alternative World Health Organization system that divides gastric cancer into papillary, tubular, mucinous (colloid), and poorly cohesive carcinomas. Both classification systems enable a better understanding of histogenesis and biology of gastric cancer yet have a limited clinical utility in guiding patient therapy, especially when dealing with the molecular heterogeneity of gastric cancer [5,6]. The TNM classification is the most important tool for planning treatment in oncology and for assessing the patient's prognosis. However, even the latest edition of the TNM classification has limited power to capture the complex cascade of progression events that derived from the heterogeneous clinical behavior of GC [7].

In the past decade, much progress has been made in identifying more accurately molecular GC subtypes by gene expression profiling based on microarray technologies [8]. Such advances hold a great promise in improving prognosis and identifying more appropriate therapies [9]. High-throughput large-scale molecular profiling data provide rich information that is unobtainable from morphological or clinical examinations alone. Unprecedented whole-genome-scale data have been catalyzing and advancing the molecular subtyping approach.

**Fig. 1:**
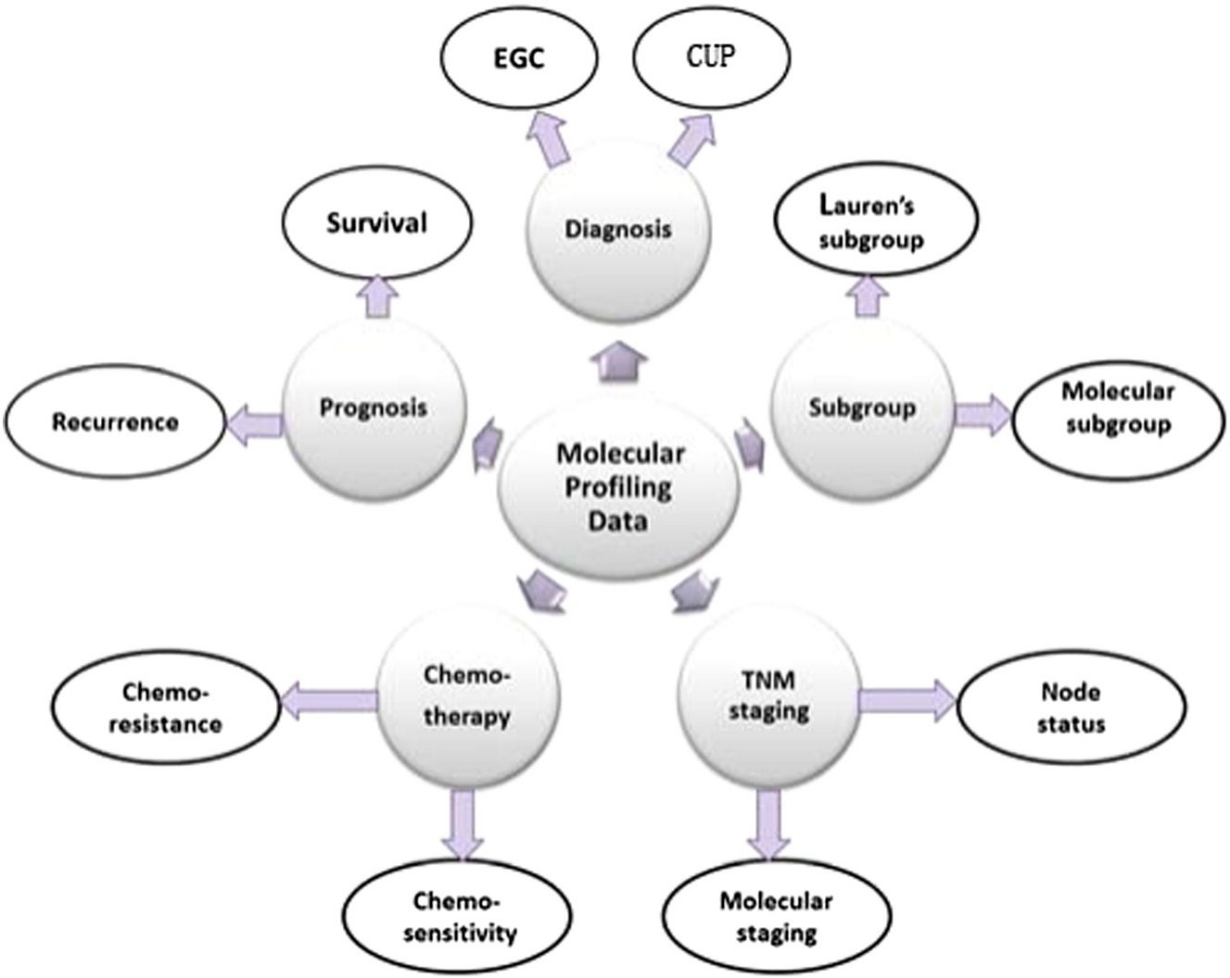
Applications of molecular profiling in diagnosis and treatment of GC. The applications of gene expression profiling in GC include diagnosis, subgroup, TNM staging, treatment, and prognosis evaluation. EGC: early gastric cancer; CUP: cancer of unknown primary site.

Here we cataloged and compared published gene expression profiling signatures in GC as well as more integrated genomic features of GC from gene expression, somatic mutation, chromosomal instability, Epstein–Bar Virus (EBV) virus infection, and DNA methylation. We highlighted the consensus patterns across these signatures, identified their associated molecular pathways, and underscored their prediction power of GC stratification and chemotherapy sensitivity. Fig. 1 outlines the contents of this review which focuses on applications of gene expression profiling in diagnosis, prognosis, and therapeutic intervention of GC.

## 2 Molecular diagnosis of GC

Gene expression signatures have successfully been identified to determine, differentiate, and categorize subtypes of GC as well as to solve some diagnostic dilemmas [8]. In early gastric cancer (EGC), tumor invasion is confined to the mucosa or submucosa regardless of the presence of lymph node metastasis or not [10]. Gene expression analysis identified a signature that differentiated EGC from normal tissue [10]. Boussioutas et al. analyzed 124 tumor and adjacent mucosa samples and explored the molecular features of gastric cancer, which could be discerned that readily defined premalignant and tumor subtypes, using DNA microarray-based gene expression profiling [11]. The identification of molecular signatures that are characteristic of subtypes of gastric cancer and associated premalignant changes should enable further analysis of the steps involved in the initiation and progression of gastric cancer. Vecchiet al. derived 1024 genes (52% up-regulated and 48% down-regulated) that were differentially expressed in 19 EGC samples when compared with 9 normal tissues [12]. The up-regulated genes are involved in cell cycle, RNA processing, ribosome biogenesis, and cytoskeleton organization, while the down-regulation genes are implicated in specific functions of the gastric mucosa (digestion, lipid metabolism, and G-protein-coupled receptor protein signaling pathway). Nam et al. [13] also identified a 973-gene signature to differentiate EGC from normal tissue using the microarray data from the matched tumor and adjacent non-cancerous tissues of 27 EGC patients [13]. They further demonstrated that the up-regulated genes in EGC tissues were correlated with cell migration and metastasis. Kim et al. demonstrated that 60 genes were gradually up or down-regulated in succession in normal mucosa, adenoma, and carcinoma samples by comparing the expression profiles of these tissues from eight patient-matched sets. Thus, molecular classification seems very promising for molecular diagnosis of EGC [14].

Both chronic gastritis (ChG) and intestinal metaplasia (IM) are involved in intermediate stage of GC, the former characterized by a mitochondria-related gene expression signature while the latter characterized by markers of proliferation [11]. Since ChG has mitochondria gene expression signature, it might be interesting to test whether such a signature is related to the metabolic subtype signature of GC [15]. Indeed, the differential expressed gene set between ChG and IM is largely overlapped with the GC metabolic signature (*P* = 0.00085, hypergeometric test).

Cancer of unknown primary site (CUP) is a well-recognized clinical disorder, accounting for 3–5% of all malignant epithelial tumors [12]. CUP can be identified based on conserved tissue-specific gene expression [16]. It has been shown that gene expression profiling can identify tissue of origin with an accuracy rate between 33% and 93% [17]. Anthony et al. applied a 92-gene CUP assay to tumor samples from patients with CUP. Fifteen of 20 cases (75%) were correctly predicted, i.e., those predicted CUPs were the actual latent primary sites that were identified after the initial diagnosis of CUP. This assay has been successfully applied to many other cancers such as breast, colorectal, and melanoma [18].

These gene signature-based methods can also be used to identify specific treatment for GC patients, i.e., targeted therapies. In a large prospective trial (*n* = 289), a gene expression signature was developed to predict the tissue of origin in most patients with CUP. The median survival time was 12.5 months for patients who received assay-directed site-specific therapy compared with the use of empiric CUP regimens. Patients whose CUP sites were predicted to have more responsive tumor types survived longer than those predicted to have less responsive tumor types [19]. These findings suggest that tumor molecular profiling can improve the treatment of patients with CUP and should be included in the standard evaluation [19].

While some great progresses have been made on molecular diagnosis based on gene expression profiling and many hospitals have built up facilities for molecular diagnosis, these technologies are still expensive and immature. Thus, reliable and cost-effective molecular diagnosis tools based on gene expression signatures have a broad development potential.

## 3 Molecular subtyping of GC

Histologically, GC shows great heterogeneity at both architectural and cytological levels and often has several co-existing tissue types such as well-developed tubular architecture and signet ring cell. The primary histopathologic classification used for GC was first described in 1965 by Lauren [20]. This classification simply divides gastric adenocarcinoma morphologically into two types: the diffuse and the intestinal types. The relative frequencies for intestinal, diffuse, and indeterminate types are approximately 54%, 32%, and 15%, respectively [21]. The intestinal type often has more well-developed tubular architecture while the diffuse type often includes poorly cohesive cells or signet ring cells [22]. Moreover, the diffuse type gastric cancer tends to carry germline mutations in genes involved in the cell adhesion protein E-cadherin; in contrast, the intestinal type is associated with atrophic gastritis, intestinal metaplasia, and *Helicobacter pylori* infection [6]. However, such classification systems do not correspond well with the degree of malignance and survivability [23]. A recent study showed that alterations of tumor-related genes did not match the histopathologic grades in gastric adenocarcinomas [5]. Furthermore, the levels of pathological differentiation are barely consistent with the prognosis ones [5,24]. The lack of a well-established grading system for gastric cancer remains as a major obstacle hindering a better clinical practice in GC.

To have better GC stratification for clinical utility, extensive efforts have been made to classify gastric tumors based on gene expression profiling. Manish et al. [25] analyzed gene expression profiling of gastric adenocarcinoma samples from 36 individual primary tumors and developed a 785-gene signature to classify gastric cancer [25]. Based on epidemiologic, histopathologic, anatomic, and molecular evidence, they classified gastric cancer into 3 subtypes—proximal non-diffuse, diffuse, and distal non-diffuse gastric cancer. An independent study shows that more than 85% of the samples were classified correctly by the 785-gene signature. The diagnostic potential of this molecular classification was further improved by using histopathologic, anatomic, and epidemiologic information.

Moreover, gene expression profiling can be utilized for the development of response to treatments. Based on the gene expression profiling data from 37 GC cell lines, Tan et al. derived a signature of 171 genes to predict two major intrinsic genomic subtypes, G-INT, and G-DIF [26]. The G-INT cell lines were significantly more sensitive to 5-fluorouracil and oxaliplatin but more resistant to cisplatin than the G-DIF cell lines. In a subsequent study, Zheng et al. identified gene expression patterns to validate three subtypes of gastric adenocarcinoma (proliferative, metabolic, and mesenchymal) [15]. Further, other levels of cancer genome features, such as genomic instability, TP53 mutations, and DNA hypomethylation, have been found in the tumors of the proliferative subtype. Cancer cells of the metabolic subtype are more sensitive to 5-fluorouracil than the other subtypes. Meanwhile, tumors of the mesenchymal subtype contain cells with characteristics of cancer stem cells and are particularly sensitive to phosphatidylinositol 3-kinase-AKT-mechanistic target of rapamycin inhibitors (PI3K-AKT-mTOR). It is very likely that this approach holds a promise toward personalized treatment.

## 4 Molecular prediction of TNM staging

The lymph node status (N classification) is a strong predictor of the outcome, and lymph node metastases usually lead to poor prognosis. However, how to predict lymph node metastasis from primary tumor is almost impossible using only pathological data. Gene expression profiling data have been utilized for this purpose.

Ken et al. developed a 92-gene signature to stratify patients with lymph node metastasis and they achieved an accuracy of 92% [27]. These genetic signatures for predicting the lymph node status can help surgeons select patients who may benefit from extended lymph node dissection. Clinica et al. screened primary gastric cancer gene expression profiles to decide whether extended lymph node dissection is necessary [28]. In this study, gene expression was first measured in frozen tumor samples obtained from 32 patients with primary gastric adenocarcinomas and then a 136 gene signature was identified to predict lymph node status. The exceptional performance (96.8% prediction accuracy) suggests that this approach can be used to tailor the extent of lymph node dissection on an individual patient basis. Cui et al. analyzed 54 pairs of matched cancer and adjacent reference tissues and identified gene expression signatures for predicting cancer grades and stages [27]. Specifically, a 10-gene signature was identified to predict early stage (stage I + II) with an accuracy of 90% and a 9-gene signature was defined to predict advanced stage cancer (stage III + IV) with an accuracy of 84%.

Moreover, gene expression-based prediction on survival can have a better performance than TNM staging. Zhang et al. reported a similar result based on a microarray study of 72 GC samples [29]. These samples were divided into two sets, a training set with 39 samples and a validation set with 33 samples. A panel of ten genes was identified in the training set as a prognostic marker that was correlated to overall survival and further verified in the validation set. Compared with the traditional TNM staging system, this ten-gene prognostic marker showed consistent prognosis results and thus was complementary to the current staging system.

## 5 Molecular prediction of response to chemotherapy

Gene expression can also be used to predict whether a GC patient responds to certain therapies. Such approaches would help provide additional predictive information for personalized treatment. Pathologic complete response to chemotherapy indicates that some tumors are extremely sensitive to chemotherapy [30]. However, it remains extremely challenging to predict chemotherapy sensitivity based on histopathological data. Several microarray assays have been developed for this purpose (Table 1).

At the early genome expression profiling stage, it has been shown that chemotherapy-sensitive tumors have significantly different gene expression than that from chemotherapy-resistant cases [31,32]. Tanaka et al. analyzed a microarray data from 19 cancer cell lines, including 2 GC cell lines, and developed a 12-gene signature to predict the response to 8 drugs (5-FU, CDDP, MMC,DOX, CPT-11, SN-38, TXL, and TXT) [33]. The signatures have the power to predict accurately not only the *in vitro* efficacy of the drugs but also GC patients' response including survival, time to treatment failure, and tumor growth to 5-FU. Nakatsu et al. established a panel of 45 human cancer cell lines (JFCR-45), including 12 stomach cancer cell lines [34]. They assessed the chemosensitivity of JFCR-45 to 53 anticancer drugs by growth inhibition experiments and built up a sensitivity database for JFCR-45 to anticancer drugs. Using these databases, they have identified gene signatures that can predict chemosensitivity of gastric cancer. Jung et al. developed G-matrix (gene expression database) and C-matrix (chemosensitivity database) from 13 gastric cancer cell lines treated with 16 anticancer agents using 22 K human oligo chips and identified an 85-gene signature be associated with chemosensitivity of gastric cancer with respect to the major anticancer drugs [35]. Recently, Ivanova et al. generated a comprehensive cohort including mRNA expression, DNA methylation, and cisplatin response data from 20 gastric cancer cell lines [36]. A panel of 291 genes was found to be differently expressed between the top four cell lines most sensitive to cisplatin and those most resistant lines. Notably, *BMP4* was overexpressed in the cisplatin-resistant cell lines. Furthermore, *BMP4* expression was significantly up-regulated (*P* = 4.53 × 10^− 5^; 2.25-fold enrichment) in 197 gastric cancer samples when compared with non-malignant gastric tissues. In primary tumors, *BMP4* promoter methylation levels were inversely correlated with *BMP4* expression, and GC patients with high *BMP4* expression in tumor exhibited significantly worse prognosis. These results suggested that *BMP4* epigenetic and expression status may represent promising biomarkers for GC cisplatin sensitivity.

The major cause of treatment failure for GC is the development of acquired resistance to chemotherapy. Gene expression signatures can be used to identify subgroups that will acquire resistance to chemotherapy. Such a strategy would provide additional predictive information for individualized treatment. Park et al. analyzed genes expression profiling of 5-FU sensitive and/or resistant GC cell lines [37]. A 13-gene signature was identified to predict response to 5-FU. Suganuma et al. identified a 23-gene signature for DDP resistance (cisplatin-resistance) by comparing the gene expression in 22 pairs of DDP-resistant tumor samples and surrounding normal tissues [38]. Similarly, Kim et al. compared the expression profiles from gastric cancer biopsy specimens obtained at a chemosensitive state with those obtained at a refractory state and identified 119 genes associated with acquired resistance to 5-FU/Cisplatin [39]. In another study, Kim et al. compared the gene expression profiling of 22 pre-CF (cisplatin and fluorouracil)-treated samples with that of the matched post-CF-treated samples and identified 72 differentially expressed genes as a signature for acquired resistance [40]. The 72-gene signature was an independent predictor for the time to progression and survival. In a similar study, they analyzed 90 gastric cancer patient samples and 34 healthy volunteers' samples using microRNA gene profiling. In total, 82 samples were used as a training set to discover candidate markers correlated to chemotherapy response, and 8 samples were used for validation. Fifty-eight microRNAs were found to be capable of discriminating patients who are likely or unlikely to respond favorably to CF therapy, suggesting that such a microRNA predictor can provide a useful guidance for personalized chemotherapy [41]. Taken together, genomic signatures derived from gene expression proofing have the capacity to connect clinical intervention especially in predicting sensitivity and resistance to specific chemotherapy regimens.

## 6 Molecular prognosis of GC

Another important function of GC Gene expression profiling is to predict which gastric cancer patients have good or poor clinical outcomes (Table 2). Many studies have shown that gene expression signatures can classify tumors into intrinsic subtypes and predict the survival of GC patients [26]. Several genomic studies also show that gene expression profiling can predict patients with a high risk for recurrence and thus can potentially improve clinical practice [42–44]. Now it is evident that gene expression techniques may significantly improve our ability to predict the risk of recurrence and to tailor the treatment for each individual gastric cancer patient.

Gene expression data in tandem with clinical information have made it possible to construct the predictive models for the outcome of gastric cancer. Yamada et al. analyzed 40 endoscopic biopsy GC samples to identify a 98-gene signature that are significantly correlated with the overall survival [45]. In particular, *PDCD6* was identified as a prognostic biomarker of GC through a multivariate analysis. Lo Nigro et al. compared gene expression profiling of 3 long-term survival cases with metastatic gastric cancer with that of 4 normal cases [46]. An 8-gene signature was identified to distinguish long survivors from the control cases. Wang et al. collected 158 gastric cancer patients, among which 33 cases were used as a training set and 125 cases for RT-PCR as a testing set [47]. A 5-gene signature was established for clinical and prognostic.

Recurrence and metastasis are the main causes for the death of GC patients. Genomic signatures have successfully been used to predict the relapse of GC. Peritoneal relapse is the most common pattern of tumor progression in advanced gastric cancer. Clinicopathological findings are often inadequate for predicting peritoneal relapse. Takeno et al. compared gene expression profiles of 38 relapse-free GC patients with those from 18 peritoneal relapse ones and developed a 22-gene signature to predict peritoneal relapse with an accuracy of 68% [48]. Cho also analyzed 65 gastric adenocarcinoma tissues and developed a risk score based on 6 genes to predict relapse of GC. This risk score was successfully tested in an independent cohort [49].

To establish prognostic index (PI) for each patient that reflects the genetic information, Kim et al. analyzed 30 pairs of gastric tumors and normal gastric tissues to develop genetic alteration score (GAS) for estimating patient's survival time by the cDNA microarray-based CGH [50]. GAS was based on 82 genes, and the prediction accuracy for recurrence was 83.33%. GAS was able to capture important genetic information for hazard rate of recurrence and distinguish a patient's recurrence status, survival status, and cancer stage status.

The development of predictive molecular models for GC treatment is still at an early stage, and those models need some substantial improvement for the use in clinical trials. High-quality studies should be conducted to develop accurate, reliable, and reproducible models for clinical practice. Only then will it be possible to use predictive models routinely to tailor GC treatment.

## 7 Comparison of predictive gene signatures in GC

Most genomic signatures were derived from data sets with a relative small sample size, raising the issue of reproducibility, especially when considering the heterogeneity nature of cancer. To examine whether those signatures are sample dependent or study specific, we systematically compared 21 gene signatures predictive of GC stages, chemotherapy response, and metastasis from 9 studies. These gene signatures had at least 70 genes and were derived from a relative larger sample population. Such selection criteria enable meaningful enrichment test.

As shown in Table 3, nine of the 21 signatures were from a recent study of GC subtypes with different responses to PI3-kinase inhibitors and 5-fluorouracil [15]. The signatures identified by Lei et al. [15] are the most comprehensive and significantly overlap with at least one signature in 7 of the other 8 studies. In this study, a cohort of 248 cases from Singapore (SG) were employed as discovering data set, with another cohort of 201 cases from Singapore and 70 cases from Australia (AU) for validation. Intriguingly, based on clinical traits, including Lauren's classification, stage of disease, a more detailed system can be obtained, involving DIF (diffused signature), INT (intestine signature), MET-sg (metabolic signature–Singapore), MET-au (metabolic signature–Australia), MES-sg (mesenchymal signature–Singapore), MES-au (mesenchymal signature–Australia), PRO-sg (proliferative signature–Singapore), and PRO-au (proliferative signature–Australia).

**Fig. 2:**
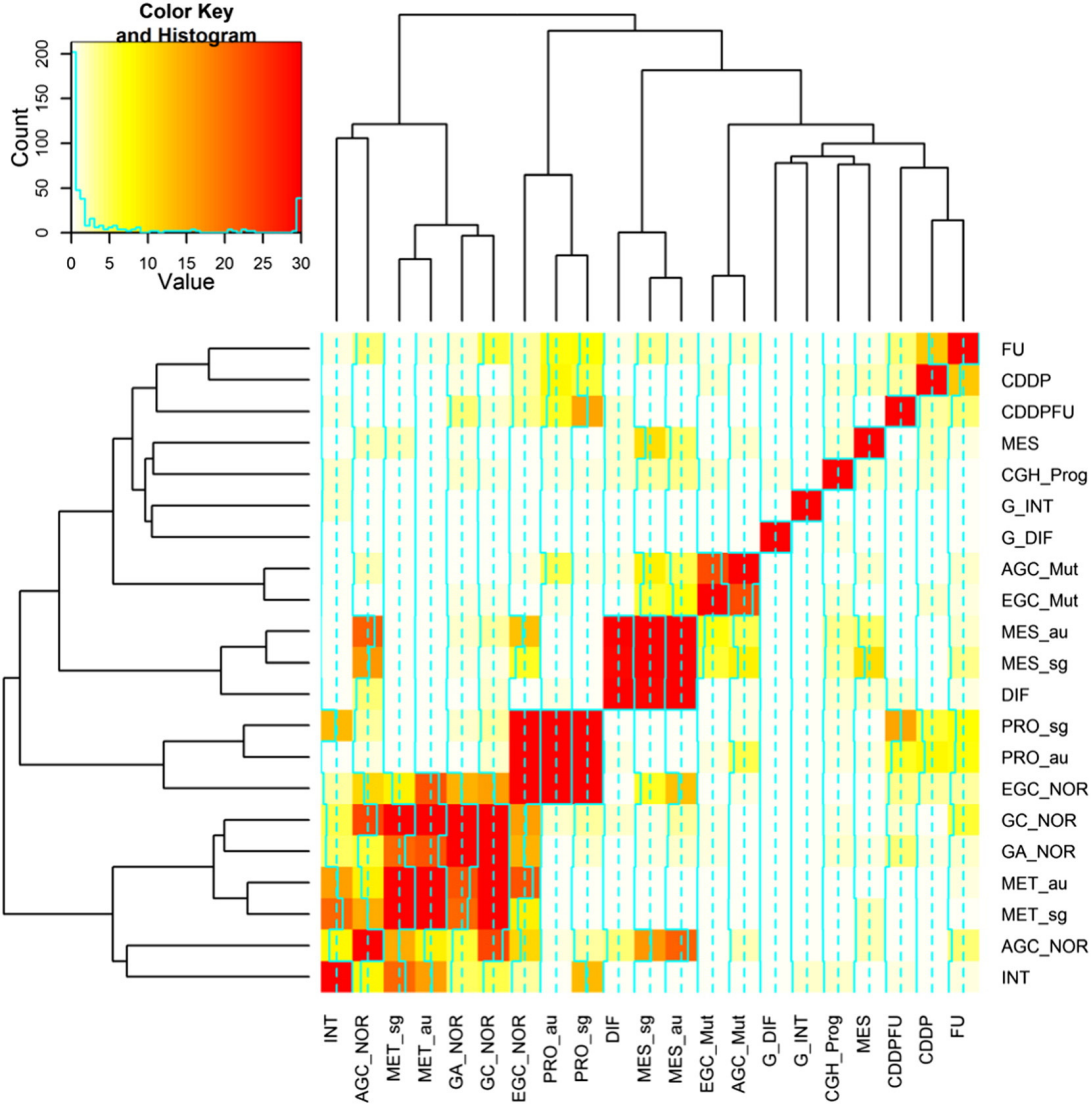
Clustering analysis of gene sets based on the significance level of overlap between signatures. Details about the signatures can be found in Table 3. The similarity between two genes signatures was determined by lg(*p* value), where *p* value was based on the hypergeometric test.

Table 4 and Fig. 2 show the overlaps between these signatures. To assess the statistical significance of an overlap between two differentially expressed gene signatures, we used the standard Fisher's exact test (FET) [51]. The INT signature significantly overlaps with MET-au and MET-sg, with FET *P* < 1.6E − 21 and *P* < 8.8E − 17, respectively. In contrast, the DIF signature overlaps more significantly with MES-sg and MES-au with FET *P* < 6.0E − 30 and *P* < 2.5E − 30, respectively, consistent to canonical Lauren's classification. The signature EGC_NOR (early gastric cancer signature) [12] highly overlaps with the proliferative signatures PRO_sg and PRO_au [15] with FET *P* < 4.2E − 42 and 8.3E − 57, respectively. Meanwhile, the signature AGC_NOR (advanced gastric cancer signature) is enriched in the MET signatures from the Singapore and Australia data sets [15] with FET *P* < 6.2E − 17 and 6.0E − 22, respectively, indicating the validity of this molecular subtype method. Moreover, the signature PRO_au [15] moderately overlaps with those chemotherapy response signatures, CDDP, CDDPFU, and FU [35,38,39,52] with FET *P* < 1.0E −8, 9.6E −7, and 7.4E −8, respectively, albeit with unknown mechanism. Interestingly, the signature EGC_NOR [12] significantly overlaps with GA_NOR [53] with FET *P* < 6.3E −15, and they share some important genes such as *RBP2*, *FHL1,* and *NME1*. *RBP2* was found to be overexpressed in GC and plays some key roles in the process of gastric carcinogenesis [54,55]. *FHL1*, a tumor suppressor gene, is involved in migration, invasion, and growth in GC due to a loss-of-function mutation [56,57]. In summary, these signatures can improve our understanding the processes from benign tumor to malignant tumor of stomach.

## 8 Integrated genomic subtyping of GC

The large-scale molecular profiling data in GC at The Cancer Genome Atlas (TCGA) provide an excellent opportunity to develop advanced molecular classifiers and predictors for GC diagnosis and treatments. Based on the TCGA data in GC, four major molecular subtypes of GC were defined, and they include EBV-infected tumors, MSI tumors, genomically stable tumors, and chromosomally unstable tumors. The molecular classification not only serves as a valuable adjunct to histopathology but also shows distinct salient genomic features providing a guide to targeted agents [58].

Recently, Cristescu et al. analyzed gene expression data of 300 primary gastric tumors to establish four molecular subtypes linked to distinct patterns of molecular alterations, disease progression, and prognosis, which included MSS/EMT subtype, MSI subtype, MSS/TP53^+^ subtype, and MSS/TP53^−^ subtype [59]. The MSS/EMT subtype includes diffuse-subtype tumors with the worst prognosis, the tendency to occur at an earlier age and the highest recurrence frequency (63%) of the four subtypes. MSI subtype contains hyper-mutated intestinal-subtype tumors occurring in the antrum, the best overall prognosis, and the lowest frequency of recurrence (22%) among the four subtypes. Patients of MSS/TP53 + and MSS/TP53 − subtypes have intermediate prognosis and recurrence rates, while the TP53-active group shows better prognosis. They also validated these subtypes in independent cohorts and showed that the four molecular subtypes were associated with not only recurrence pattern and prognosis but also distinct patterns of genomic alterations. These subtypes can provide a molecular subtyping framework for preclinical, clinical, and translational studies of GC.

Whole genomic sequencing has the capacity to define subtyping of GC at the DNA level. We summarized the recent advances in this direction in Table 5. These mutation signatures are anticipated to open a new avenue for targeted GC therapy.

## 9 Biological functions underlying gene signatures in GC

Gene signatures derived from gene expression profiling of tumor samples not only allow us to stratify GC cases as classifier but also enable us a better understanding of the underlying biological process and molecular pathways. Thus, we further examined the enrichment of gene signatures by intersecting these signatures with those gene sets listed in the MSigDB database [60].

**Fig. 3:**
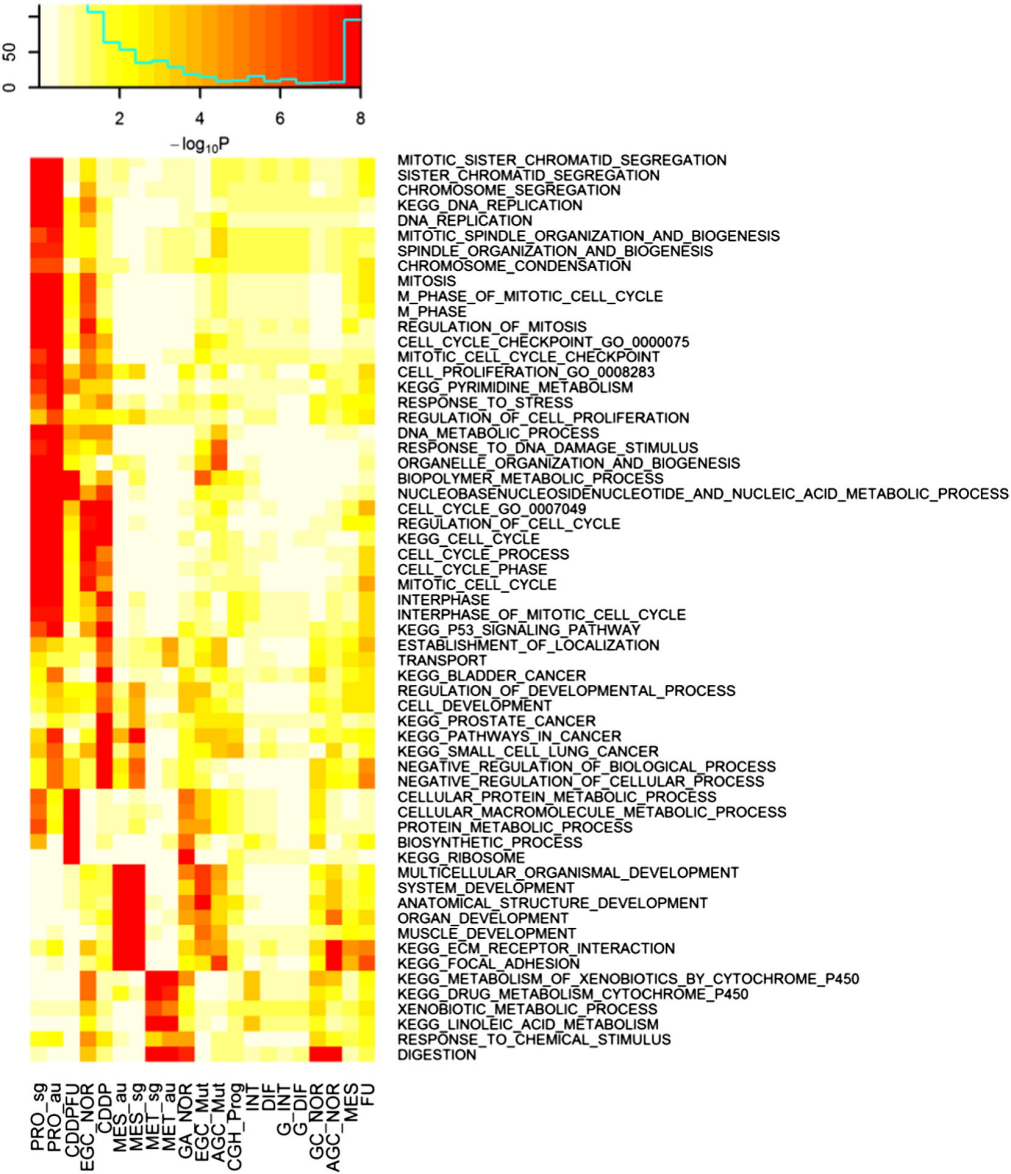
Enrichment of MSigDB and gastric specific gene sets in the GC gene signatures. The c2 and c5 sets include biological processes and KEGG from MSigDB version 5.0 were employed for enrichment of the analysis of gene sets listed in Table 3.

The c2 and c5 sets in MSigDB version 5.0, including biological processes and KEGG pathways, respectively, were tested for enrichment in the gene signatures listed in Table 3. The result was represented by the heat map in Fig. 3. Many cancer-associated molecular pathways have been captured in terms of highly overlapped with GC gene signatures. Notably, the digestion and pyrimidine signaling pathways highly enriched in many signatures, indicating they are GC specific and related to inflammation.

## 10 Summary and prospective

The findings from the analysis of gene expression data in GC has a significant impact on our understanding of GC biology by bringing the concept of the heterogeneity of GC to the forefront of GC research and clinical practice [8]. Gene expression profiling technology will enable clinicians not only to estimate the likelihood that certain therapies will be beneficial but also to determine when and how to modify treatment options. More informed decision making will ultimately enable increased rates of response and survival [9,15,26,40,47]. High-throughput molecular techniques will not replace conventional clinical and pathological evaluation to classify GC but rather serve as an adjunct to known clinical methods.

Many gene signatures have been developed, but there is little overlap between those gene lists, and the reproducibility is usually very poor [8]. The poor reproducibility of these models is due to many intrinsic problems associated with heterogeneity in patient populations and tumors and microarray-based approaches. First, patient populations and treatments are diversified: different patient demographics and varied treatment regimens lead to variations into predictive classifiers. Distributions of age, race, and gender have a significant impact on molecular profiling [8]. Therefore, it is difficult to compare expression data from different treatment plans, and such inconsistency limits our ability to develop robust predictive molecular models. Second, the selection of samples is not consistent. Tumors vary in their composition and are highly heterogeneous for stromal and cancerous components. Micro-dissection is often used to ensure that a pure tumor cell population can be profiled. Intriguingly, stromal signatures have been shown to be informative for predicting chemo sensitivity, recurrence, and outcome [61]. Third, there are different profiling platforms and statistical analysis approaches. Biases in profiling platforms and analytic approaches further complicate the reproducibility. Therefore, it is essential that studies are designed and carried out thoughtfully to gain the most appropriate and relevant information. Fourth, there lacks large-scale independent validation. While many studies have used molecular profiling data to develop predictors for predicting treatment response and prognosis in GC, those models share a very limited number of genes. Thus, reliability and reproducibility of microarray data remain questionable unless their performance is confirmed at a relatively large scale.

Recently, several gene microarray-based tools have been commercially developed for clinical use in breast cancer [62,63]. Now the clinical practice of predictive arrays in gastric cancer is falling behind relative to breast cancer. A lot of factors contribute to this lag-behind, but perhaps the first and foremost is the drastically greater volume of research into predictive medicine in breast cancer [63]. Comparing to breast cancer, there are much less common and ongoing controversies in optional multimodality therapy in GC [52]. With more advanced technologies and expanding knowledge from a multitude of existing studies, more accurate subtypes of GC are likely to be teased out from the existing groups. Better characterization of genetic subtypes of gastric cancer may reduce the biological variation and allow the generation of more robust predictive signatures for individual tumor subtypes. Only then will it be possible to apply predictive genomics to clinical practice.

The development and improvement of gene expression assays have led to some major breakthroughs in GC research, which will have the potential to influence the clinical treatment of patients. Although there are significant challenges in implementing genomic medicine in GC [8,64], the future genomic medicine will dramatically reshape how the disease is characterized and defined, how medicine is managed to patients, and how patients are given tailored therapies. The large-scale molecular profiling data in GC at The Cancer Genome Atlas (TCGA) provide an excellent opportunity to develop advanced molecular classifiers and predictors for GC diagnosis and treatment.

## Author contributions

BZ conceived the idea and supervised the data analysis. BZ and XL wrote the manuscript. XL, YZ, and WS performed data analysis. All authors read, edited, and approved the final manuscript.

## Figures and Tables

**Table 1:** Gene expression profiling associated with sensitivity or resistance to anticancer drugs in GC.

Signature	Samples	Drugs	Result	Reference
NA	Three sensitive and one resistant GC cell line	Cisplatin	Patterns of gene expression alteration after exposure to cisplatin/5-flu	Wesolowski and Ramaswamy[62]
250 genes	Ten chemoresistant and 4 parent GC cell lines	Cisplatin	Offered gene information with acquired resistance	Kang et al. [65]
13 genes	Eight GC cell lines	5-FU	Provided biomarkers for 5-FU sensitivity/resistance	Park et al. [37]
23 genes	35 GC cases	5-FU	Gave information regarding chemoresistance factors	Suganuma et al. [38]
69 genes/5 flu and 45 genes/cisplatin	Three GC cell lines	5-FU	Predicted responses to 5-flu	Ahn et al. [66]
39 genes	NA	5-FU	39-gene signature with 5-FU resistance	Szoke et al. [67]
119 genes	Seven GC cases	5-FU/cisplatin	Distinguished chemosensitive state from the refractory state	Kim et al. [39]
four genes	Three cell lines and 37 GC	Paclitaxel	Provided new markers for resistance to paclitaxel	Murakami et al. [68]
NA	30 cancer cell lines	5-FU	constructed profiles of resistance against each chemotherapy agent	Gyorffy et al. [69]
NA	45 cancer lines including 12 GC cell lines	53 drugs	Established a sensitivity database for JFCR-4andatabase of the EGF	Nakatsu et al. [34]
12 genes	19 cell lines and 30 GC	8 drugs	Developed prediction models of the 8 anticancer drugs	Tanaka et al. [33]
85 genes	13 GC cell lines	16 drugs	Acted as markers for chemosensitivity in chemo-naive GC patients	Jung et al. [35]
seven genes	20 GC cases and 19 GC validation	Doxorubicin	Predicted the response of GC to doxorubicin	Hao et al. [70]
MRP4	One GC cell line(SGC7901)	Cisplatin	MRP4 is a DDP resistance candidate gene	Yan-Hong et al. [71]
NA	Three GC cell lines	Parthenolide	Enhanced chemosensitivity to paclitaxel in the treatment	Itsuro et al. [72]
NA	Three GC cell lines	Vorinostat	Vorinostat improved the outcomes of GC patients	Sofie et al. [73]
NA	Three GC cell lines	Metformin	Metformin inhibited GC cell and proliferation	Kiyohito et al. [74]

**Table 2:** Gene expression profiling for GC prognosis.

Signature	Data set	Results	Reference
Three oncogenic pathways	25 GC cell lines of discover set and 300 cases of validation set	3 oncogenic pathway combinations predicted clinical prognosis	Ooi et al. [75]
Two genomic subtypes (G-INT and G-DIF)	37 GC cell lines of discover set and 521 cases of validation set	Associated with patient survival and response to chemotherapy	Tan et al. [26]
98 genes	40 cases of discover set and 19 cases of validation set	Predicted the overall survival	Yamada et al. [45]
Eight genes	Seven cases and four cases control	Had a predictive role in survival of metastatic patients	Lo Nigro et al. [46]
82 genes signature	30 pairs of gastric mucosa and cancer	Reflected the genetic information for hazard rate of recurrence	Kim and Rha [50]
Five genes	33 cases of discover set and 125 cases of validation set	Independent prognostic factors for overall survival	Wang et al. [47]
Four genes	48 cases	Predicted surgery-related survival	Xu et al. [76]
Six genes	65 cases of discover set and 96 cases of validation set	Predicted the likelihood of relapse after curative resection	Cho et al. [49]
Two genes	Seven cases recurrence and four cases without recurrence	Acted as new prognostic biomarkers in predicting recurrence risk	Yan et al. [77]
hsa-miR-335	74 cases of discover set and 64 cases of validation set	Had the potential to recognize the recurrence risk	Yan et al. [78]
Three miRs	45 cases	Predicted of recurrence of GC	Brenner et al. [79]
Two miRs	65 cases of discover set and 57 cases of validation set	As a predictor of disease progression	Zhang et al. [80]
Five microRNA	164 cases and 127 normal control	Expression levels of miRNAs indicated tumor progression stages	Kim and Chung [81]
*CD26*	32 cases of GIST	Played an important role in the progression of GISTs and serve as a therapeutic target	Yamaguchi et al. [82]
*CCL18*	90 cases of discover set and 59 cases of validation set	As an independent prognostic indicator	Leung et al. [83]
Three genes	18 cases of discover set and 40 cases of validation set	Predicted surgery-related outcome	Chen et al. [84]
22 genes	56 cases of discover set and 85 cases of validation set	Be useful in prospective prediction of peritoneal relapse	Takeno et al. [48]
*CD9*	senveGISTs of discover set and 117 GISTs of validation set	As potent prognostic markers in GIST	Setoguchi et al. [85]
29 genes	60 cases of discover set and 20 cases of validation set	Improved the prediction of recurrence in patients	Chen et al. [84]

**Table 3:** Descriptions of signatures used for a systematic comparison in Table 4.

Signature	Size	Description
CGH_Prog [50]	70	Prognosis signature of array CGH probes
DIF [15]	78	Expression signature of diffused type
G_DIF [26]	79	Diffusion type signature
MES [15]	89	Mesenchymal signature
G_INT [24]	91	Gastric intestine signature
INT [15]	91	Intestine signature
FU [35,38,39,52]	131	5 Fu response signature
CDDP [35,38,39,52]	224	Cisplatin response signature
GA_NOR [50]	264	Gastric adenoma signature
AGC_NOR [11]	309	Advanced gastric cancer signature
MET_au [15]	315	Metabolic signature–Australia
GC_NOR [50]	364	Gastric carcinoma signature
CDDPFU [35,38,39,52]	444	5 Fu and cisplatin response signature
AGC_Mut [83,84]	446	Advanced gastric cancer mutation signature
MET_sg [15]	736	Metabolic signature–Singapore
EGC_NOR [11]	815	Early gastric cancer
PRO_au [15]	854	Proliferative signature–Australia
EGC_Mut [85]	857	Early gastric cancer mutation signature
MES_au [15]	1398	Mesenchymal signature–Australia
PRO_sg [15]	2244	Proliferative signature–Singapore
MES_sg [15]	2920	Mesenchymal signature–Singapore

Abbreviations and source literatures are listed in the first column of the table.

**Table 4:**
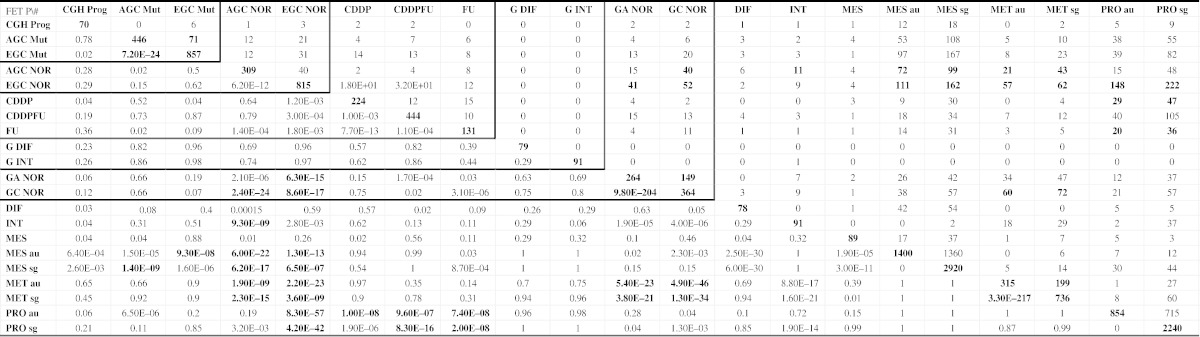
Overlap between the gene signatures specified in Table 3. The diagonal of the matrix below represent the number of genes in each signature. The elements in the upper-right panel represent the number of genes shared by two signatures while those in the lower-left panel represent the corresponding *p* values computed based on the hypergeometric test.

**Table 5:** Integrative gastric subtyping studies including The Cancer Genome Atlas (TCGA), the Asia Cancer Research Group (ACRG), and diffusion gastric adenocarcinoma (DGC).

System	Molecular subtypes	Sample size	Percentage	Reference
TCGA		295		
	EBV positive		8.81	TCGA [58]
	MSI high		21.69	
	GS		19.66	
	CIN		49.83	
ACRG		300		Cristescu et al. [59]
	MSS/TP53 +		35.70	
	MSS/TP53 −		26.30	
	MSS/EMT		15.30	
	MSI		22.70	
genomic alteration				Deng et al. [86]
	FGFR2		9.00	
	KRAS		9.00	
	EGFR		8.00	
	ERBB2		7.00	
	MET		37.00	
DGC-RHOA-Japan		98		Wang [87]
	RHOA +		14.70	
	RHOA −		85.30	
DGC-RHOA-HKU		87		
	RHOA +		25.3	Kakiuchi [88]
	RHOA −		74.7	
Mutation signature		49		Wong et al. [87]
	TpT		36.73	
	CpG		NA	
	TpCp[A/T]		NA	
